# Supplementary material for: Transcriptome Sequence Reveals Candidate Genes Involving in the Post-Harvest Hardening of Trifoliate Yam Dioscorea dumetorum
Source: Plants (Basel). 2021 Apr 16;10(4):787. doi: 10.3390/plants10040787 (PMC8074181; doi:10.3390/plants10040787)
Supplement: Supplementary file 1 [file plants-10-00787-s001.zip › plants-1130765-proofed suppl/Plants_Suppl/File_S1.pdf]

Accessions	Conditions	Total cleaned reads	Total (%)	Uniquely mapped reads (%)	Total (%)
Bangou 1	4MAE	17893940	56.61	36.51	45.13
Bangou 1	4MAE	18949046	93.49	60.09	69.42
Bangou 1	4MAE	20612907	92.51	58.2	69.05
Bangou 1	AH	21284551	96.13	59	74.38
Bangou 1	AH	20481906	91.77	53.96	70.26
Bangou 1	AH	17929078	93.36	53.29	78.56
Bangou 1	3DAH	19902140	95.5	56.66	74
Bangou 1	3DAH	24032161	93.56	51.76	78.23
Bangou 1	3DAH	14752529	86.88	52.2	70.09
Bangou 1	14DAH	18279097	77.73	45.16	63.81
Bangou 1	14DAH	20344788	91.64	56.5	74.26
Bangou 1	14DAH	4536123	93.3	58.73	69.95
Bayangam 2	4MAE	53929557	96.63	63.53	71.97
Bayangam 2	4MAE	22569645	91.81	60.56	71.45
Bayangam 2	4MAE	24937580	88.9	57.83	65.31
Bayangam 2	AH	21708627	82.96	52.63	61.75
Bayangam 2	AH	23997962	97.29	53.7	80.6
Bayangam 2	AH	31335502	94.09	49.7	78.78
Bayangam 2	3DAH	24284434	94.56	54.66	72.63
Bayangam 2	3DAH	22565017	93.62	56.11	76.8
Bayangam 2	3DAH	20803207	96.21	58.49	74.35
Bayangam 2	14DAH	27593312	90.11	55.12	72.04
Bayangam 2	14DAH	18519729	95.9	62.63	71.94
Bayangam 2	14DAH	19713286	96	60.25	70.93
Fonkouankem 1	4MAE	22166474	94.25	62.53	71.25
Fonkouankem 1	4MAE	12111303	84.48	55.59	61.48
Fonkouankem 1	4MAE	20286805	85.76	56.75	61.04
Fonkouankem 1	AH	13126435	93.29	53.1	74.92
Fonkouankem 1	AH	19560305	92.51	55.3	73.54
Fonkouankem 1	AH	13307694	93.43	56	70.66
Fonkouankem 1	3DAH	22500916	92.21	56.99	71.71
Fonkouankem 1	3DAH	17371457	96.19	56.96	74.36
Fonkouankem 1	3DAH	11617175	95.44	57.81	67.16
Fonkouankem 1	14DAH	20060449	93.44	62.04	68.06
Fonkouankem 1	14DAH	15829240	86.93	56.82	71.64
Fonkouankem 1	14DAH	15279090	86.98	56.88	63.02
Ibosweet 3	4MAE	16672162	93.04	59.72	68.71
Ibosweet 3	4MAE	13924548	91.2	58.68	68.4
Ibosweet 3	4MAE	30873744	91.99	57.53	70.07
Ibosweet 3	AH	7881301	93.36	55.37	73.71
Ibosweet 3	AH	14683717	94.95	56.98	75.31
Ibosweet 3	AH	14787799	95.71	61.83	72.2
Ibosweet 3	3DAH	14413912	95.55	59.9	73.23
Ibosweet 3	3DAH	21994748	94.17	56.54	73.06
Ibosweet 3	3DAH	17962629	95.22	55.32	73.62
Ibosweet 3	14DAH	15590855	91.61	58.23	68.25
Ibosweet 3	14DAH	21780220	84.31	52.77	65.4
Ibosweet 3	14DAH	18583946	95.04	60.53	72.64
