# Supplementary material for: Transcriptome Sequence Reveals Candidate Genes Involving in the Post-Harvest Hardening of Trifoliate Yam Dioscorea dumetorum
Source: Plants (Basel). 2021 Apr 16;10(4):787. doi: 10.3390/plants10040787 (PMC8074181; doi:10.3390/plants10040787)
Supplement: Supplementary file 1 [file plants-10-00787-s001.zip › plants-1130765-proofed suppl/Plants_Suppl/File_S11.docx]

| **NUMBER** | **GENE ANOTATION** | **CONTIG NUMBER** | **SEQUENCE** |
| --- | --- | --- | --- |
| CS29 | *LHCB1* | contig206.g11-F | GGCCCAAGACTGTTTCTGGA |
| CS30 |  | contig206.g11-R | TCACGGTTCTTGGCGAAAGT |
| CS33 | *CESA* | contig60.g53-F | GACGATGGGGAATTCGCTGA |
| CS34 |  | contig60.g53-R | AGCACAACAATGGTGGGAGT |
| CS35 | *LACCASE* | contig199.g1672-F | CCACTGCACTCCTACAAGCA |
| CS36 |  | contig199.g1672-R | CTGCAAGTTTGCTGGACCAC |
| CS39 | *MYB46* | contig267.g494-F | AGTTCTTGGCAACAGGTGGT |
| CS40 |  | contig267.g494-R | CCGGCGACTCGCTGTTATTA |
| CS19 | *ACTIN* | ACT-F | CTCATTGATCGGCATGGAAGC |
| CS20 |  | ACT-R | GGGGAACATAGTTGAACCACCAC |
