# Supplementary figures and images for: Transcriptome Sequence Reveals Candidate Genes Involving in the Post-Harvest Hardening of Trifoliate Yam Dioscorea dumetorum
Source: Plants (Basel). 2021 Apr 16;10(4):787. doi: 10.3390/plants10040787 (PMC8074181; doi:10.3390/plants10040787)

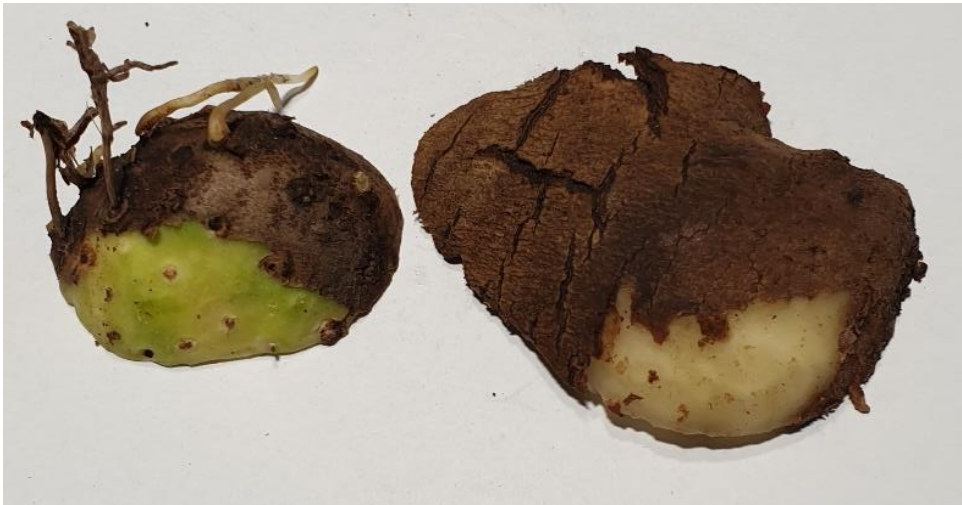

Supplement: Supplementary file 1 [file plants-10-00787-s001.zip › plants-1130765-proofed suppl/Plants_Suppl/File_S10.pdf]

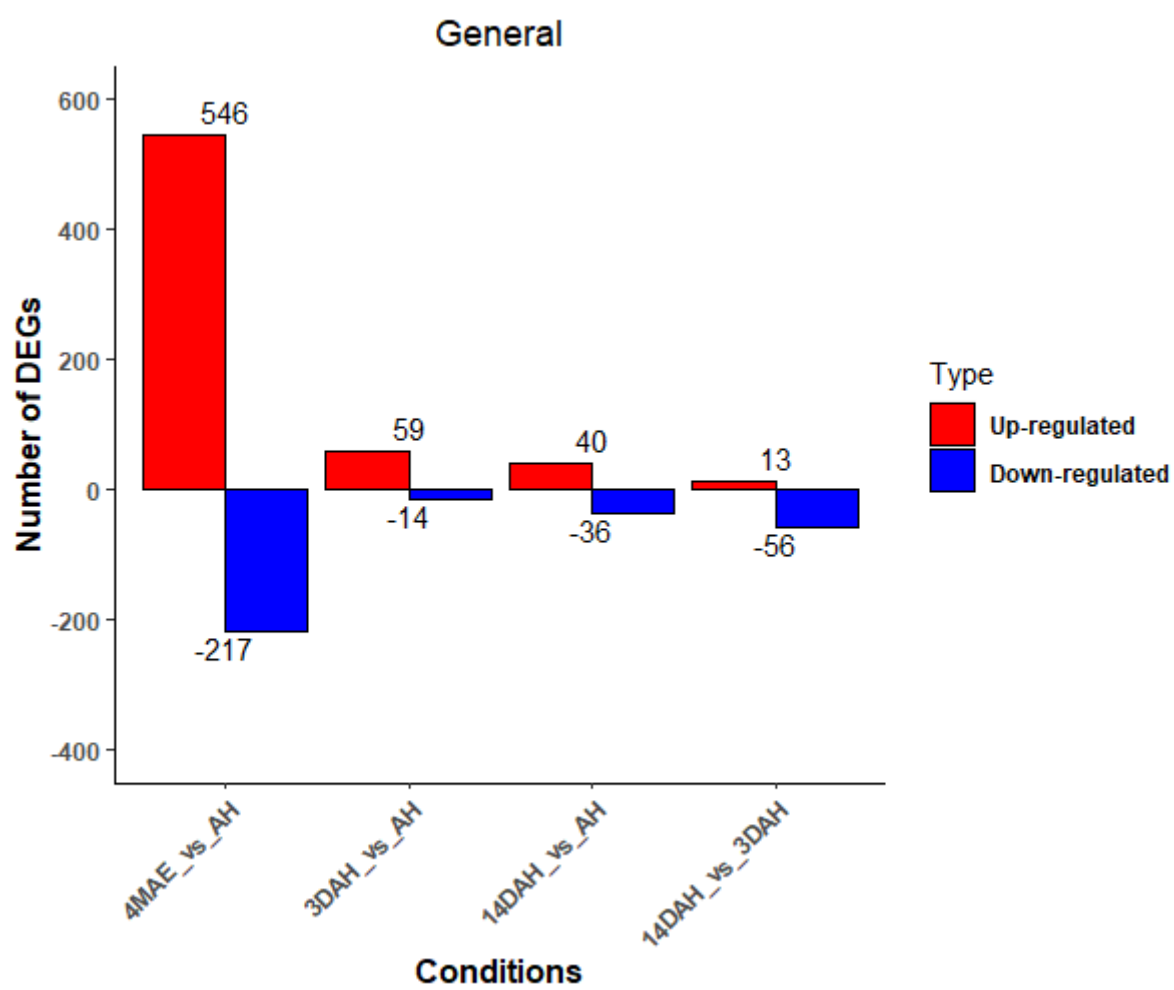

Supplement: Supplementary file 1 [file plants-10-00787-s001.zip › plants-1130765-proofed suppl/Plants_Suppl/File_S3.pdf]

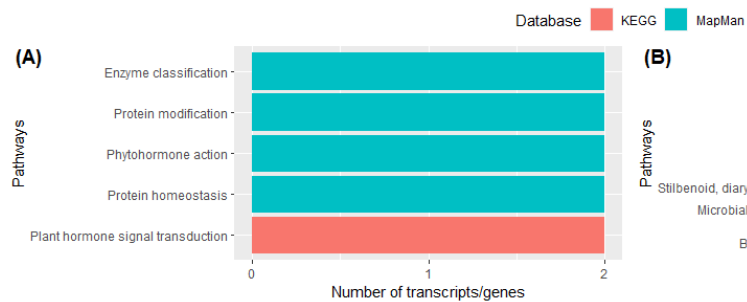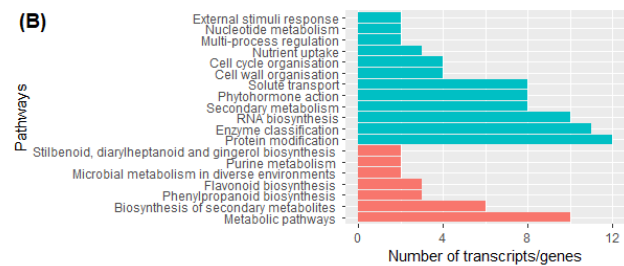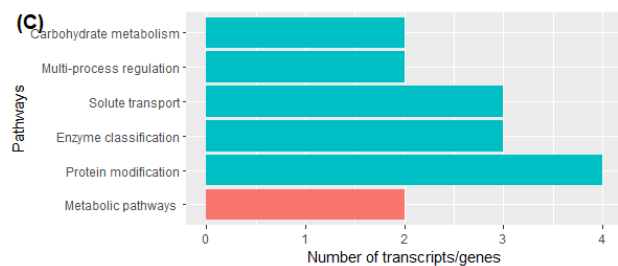

Supplement: Supplementary file 1 [file plants-10-00787-s001.zip › plants-1130765-proofed suppl/Plants_Suppl/File_S9.pdf]
